# Proton Conductivity through Polybenzimidazole Composite Membranes Containing Silica Nanofiber Mats

**DOI:** 10.3390/polym11071182

**Published:** 2019-07-14

**Authors:** Jorge Escorihuela, Abel García-Bernabé, Alvaro Montero, Andreu Andrio, Óscar Sahuquillo, Enrique Gimenez, Vicente Compañ

**Affiliations:** 1 Departamento de Termodinámica Aplicada (ETSII), Universitat Politècnica de València, Camino de Vera s/n, 46022 Valencia, Spain; 2 Departament de Química Orgànica, Universitat de València, Av. Vicent Andrés Estellés s/n, Burjassot, 46100 Valencia, Spain; 3 Departament de Física Aplicada, Universitat Jaume I, 12080 Castelló, Spain; 4 Instituto de Tecnología de Materiales, Universitat Politècnica de València, Camino de Vera s/n, 46022 Valencia, Spain

**Keywords:** fuel cells, proton exchange membrane, polymer, polybenzimidazole, silica, nanofibers, electrospinning, proton conductivity, electrochemical impedance spectroscopy

## Abstract

The quest for sustainable and more efficient energy-converting devices has been the focus of researchers′ efforts in the past decades. In this study, SiO_2_ nanofiber mats were fabricated through an electrospinning process and later functionalized using silane chemistry to introduce different polar groups −OH (neutral), −SO_3_H (acidic) and −NH_2_ (basic). The modified nanofiber mats were embedded in PBI to fabricate mixed matrix membranes. The incorporation of these nanofiber mats in the PBI matrix showed an improvement in the chemical and thermal stability of the composite membranes. Proton conduction measurements show that PBI composite membranes containing nanofiber mats with basic groups showed higher proton conductivities, reaching values as high as 4 mS·cm^−1^ at 200 °C.

## 1. Introduction

The serious environmental problems associated with the use of fossil fuels have focused the interest in developing environmentally benign devices capable of producing energy. Fuel cells (FCs) are electrochemical devices that cleanly and efficiently produce electrical energy from the chemical energy of hydrogen or another fuel [1]. The quest for novel proton conducting membranes has gained increasing attention in the past decades due to their potential applications in fuel cells [2]. In a common polymeric-exchange membrane fuel cell (PEMFC), the polymeric electrolyte membrane constitutes the fundamental element of the device, as it is responsible of the proton conductivity, and consequently, most studies have been focused in analyzing this component. Among the different varieties of PEMs used along the past, those based on perfluorosulfonic acid (PFSA) membranes, such as Nafion©, have been by far the most investigated electrolyte materials mainly due to their high chemical and mechanical stability and high proton conductivity under high humidity conditions [3]. Despite they have been widely used along the past decades, the major drawback of PFSA-based membranes is their low proton conductivity at temperatures above 90 °C [4], which limits their use for low temperature PEMFC applications. As an alternative to low-temperature proton exchange membrane fuel cells (LT-PEMFCs), high-temperature proton exchange membrane fuel cells (HT-PEMFCs), which operate at temperatures between 120 and 200 °C have received increasing attention as they offer several benefits over LT-PEMFCs, such as improved tolerance to catalyst poisoning, superior kinetics of electrochemical reactions, facile water and heat management, higher tolerance of fuel impurities, high waste heat utilization, and simplified system design [5]. Among the different variety of PEMs operating at elevated temperatures, those based on polybenzimidazole (PBI) have emerged as promising candidates for HT-PEMFCs [6].

PBI is a synthetic polymer with and excellent chemical and thermal stability, which exhibit high proton conductivities at high temperatures when doped with different acidic doping agents, generally phosphoric acid (PA) [7]. The main drawbacks of using PBI-PA composite membranes are the reduction of mechanical strength, the non-desired leaking of PA from the membrane, as well as the catalyst degradation at elevated temperature [8]. Consequently, many efforts have been diverged to develop new alternative approaches to increase the proton conductivity in PBI-based membranes. In recent years, the preparation of mixed matrix membranes (MMMs) has emerged as a promising strategy in the preparation of PEMFCs with increased proton conductivity [9]. This approach combines the use of an organic polymer with an inorganic agent acting as a filler. In this regard, different fillers have been explored in PBI membranes for this purpose, including metalcarborane salts [10], graphene and graphite oxide [11,12], multiwalled carbon nanotubes [13,14], molecular organic frameworks (MOFs) [15,16], ionic liquids [17], phosphotungstic acid [18], and silica-based materials among others [19,20,21].

Electrospun nanofibers have attracted considerable attention in fields such as in catalysis [22], drug delivery systems [23], tissue engineering [24], and in recent years, they have been explored as fillers in the fabrication of PEMFCs, such as Nafion [25,26,27], SPEEK [28,29,30], PVA [31] and chitosan membranes. In this regard, inorganic SiO_2_ nanofibers have been successfully used as reinforcement agents for the fabrication of proton exchange membranes. To this end, Wang and co–workers prepared SPEEK and chitosan membranes containing SiO_2_ nanofibers with different functional groups to fabricate highly conductive PEMs. In their study, highest proton conductivities were observed for SPEEK membranes containing SiO_2_ nanofibers with basic amino groups, with values up to 0.094 S·cm^−1^ at 65 °C and 100% RH. However, under the same operating conditions SiO_2_ nanofibers containing acidic sulfonic groups only reached conductivities up to 0.066 S·cm^−1^ [30]. In another work, Lee et al. reported on the preparation of a composite membrane of silica/SPEEK nanofiber mat impregnated with Nafion, whose proton conductivity reached values of 0.077 S·cm^−1^ at 90 °C and 100% RH. In a recent study, Zhuang and co-workers fabricated Nafion membranes with SiO_2_ nanofibers, which were functionalized with (3-aminopropyl)triethoxysilane (APTES) and subsequent EDC/NHS coupling with amino acids bearing different polar groups. The study showed that hybrid membranes reinforced with electrospun nanofibers bearing cysteine amino acid with thiol groups, yielded conductivities up to 0.242 S·cm^−1^ at 80 °C and 100% RH [26]. Despite high conductivities that have been reached for nafion membranes with silica-based nanofibers, these composite membranes are not suitable for their application in HT-PEMFCs, where operating temperature is above 120 °C.

**Figure 1:**
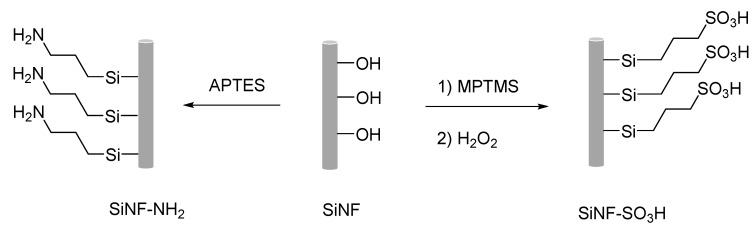
Schematic representation of silica nanofibers modification for preparing nanofibers containing acidic (SiNF–SO_3_H) or basic groups (SiNF–NH_2_).

Herein, a series of silica nanofiber mats containing different functional groups (neutral, acidic, or basic groups) were fabricated by electrospinning and functionalized with organosilane compounds in order to introduce basic or acidic groups (Figure 1). After chemical modification using silane chemistry, these nanofiber mats were embedded into a PBI matrix and their physicochemical properties were evaluated in terms of dimensional stability, water uptake (WU), thermal and oxidative stability and proton conductivity. The incorporation of these nanofibrous fillers into the polymeric matrix showed a significant improvement in the chemical and thermal stability of the composite membranes. The proton conductivity of the composite membranes was evaluated by electrochemical impedance spectroscopy (EIS) and activation energies were calculated. The results indicate that PBI composite membranes containing nanofiber mats with basic groups showed higher proton conductivities as compared as the acidic or neutral nanofibers, reaching values as high as 4 mS·cm^−1^ at 200 °C.

## 2. Materials and Methods

### 2.1. Materials

*Meta*-Polybenzimidazole (PBI) with a purity higher than 99.95% and molecular weight around 51,000 (with the molecular formula: (C_20_H_12_N_4_)*_n_*) was purchased from Danish Power Systems (Danish Power Systems, Kvistgaard, Denmark). *N*,*N*–Dimethylacetamide (DMAc, 99.5% extra pure), (3-aminopropyl) triethoxysilane (APTES, 99%), (3-mercaptopropyl) trimethoxysilane (MPTMS, 97%), tetraethyl orthosilicate (TEOS, 99.9%), ethanol, hydrochloric acid (HCl, 37%), ammonia water (25%), H_2_O_2_ (30% *v*/*v*) were purchased from Sigma-Aldrich (Sigma-Aldrich Química SL, Madrid, Spain). All chemicals were of analytical grade and used as received without any purification.

### 2.2. Preparation of Silica Nanofiber Mats

The silica solution used for the electrospinning process was prepared from a TEOS solution prepare as follows. Initially, TEOS was added to a mixture of ethanol, deionized water and HCl (in a molar ratio, 2:2:0.01) followed by stirring and heating at 80 °C until the volume of the mixture decreased by 3/8 of the initial volume (approximately after heating during 4 h). Afterwards, the same weight of dimethylformamide (DMF) was added to the TEOS solution, and the mixture was thoroughly mixed for 1 h under stirring at 60 °C. The obtained solution was subsequently used in the electrospinning process in a horizontal set-up. For that, the solution was loaded in a plastic disposable syringe with a 0.7 mm of internal diameter (I.D.) with a needle, and then pumped through a Teflon tube with a syringe pump TYD01 (Lead Fluid Technology, Heibei, China) at a constant rate of 0.0125 mL·min^−1^. A high voltage power supply DW-N503-4ACDE (Dongwen High Voltage Power Supply, Tianjin, China) which provided 15 kV potential was connected to the needle. The needle-to-collector distance was fixed at 15 cm and the rotating speed of the collector was set at 100 rpm. Under these operating conditions, silica nanofibers were obtained via electrospinning. The collected nanofibers were then air dried for 10 h and further dried in a vacuum oven at 80 °C for 10 h to remove residual solvent. The chemical modification of silica nanofiber mats was performed as previously described using alkoxysilane chemistry [30].

### 2.3. Preparation of PBI Solution

The 10 wt % PBI solution in DMAc was prepared as follows. Initially, LiCl as a stabilizer was dissolved in DMAc under vigorous stirring at 50 °C for 30 min to give a homogeneous solution containing LiCl at 0.1 wt %. Next, 10 g of PBI powder were dissolved in 90 g of LiCl solution in DMAc and heated under reflux at 120 °C for 6 h to give a final PBI solution with a 10 wt % PBI content. The prepared solution had a viscosity of 0.5 Pa·s at 25 °C.

### 2.4. Preparation of the Composite PBI@SiNF Membranes

Composite membranes were prepared by PBI impregnation into electrospun silica nanofiber mats. For that, the corresponding silica nanofiber mats were embedded in the 10 wt % PBI solution. Then, the solution was cast onto a glass slide and dried at 70 °C for 10 h, then at 140 °C for 10 h, and finally at 120 °C under vacuum overnight.

### 2.5. Characterization

For electrospinning preparation of silica nanofibers, a Super ES-2 model E-Spin Nanotech electrospinning machine was used (E-Spin Nanotech, Kanpur, India). For technical details of scanning electron microscopy (SEM, Zeiss, Oberkochen, Germany), attenuated total reflection Fourier transform infrared (ATR–FTIR, Jasco Spain, Madrid, Spain) spectroscopy, thermogravimetric analysis (TGA, Waters Cromatografia, S.A., Division TA Instruments, Cerdanyola del Valles, Spain), water uptake (WU), oxidative stability (OS) by Fenton’s test, mechanical properties and electrochemical impedance spectroscopy (EIS, Novocontrol Technologies, Hundsangen, Germany) see other previously published procedures [15,17,32].

## 3. Results and Discussion

### 3.1. Preparation and Characterization of Silica Nanofibers

The electrospinning technique is a low-cost and efficient method for generating nanofibers with tunable properties which are of interest for a wide range of applications [33]. This technique has been largely used to obtain polymeric nanofibers from polymer solutions; however, its use has not been so widespread in the production of electrospun ceramic nanofibers. In most cases, it has been successfully achieved by adding polymers or gelling agents in the electrospinable solution [34]. Subsequently, the nanofibers need to be subjected to high temperature heat treatment in order to calcine or remove all organic components. In recent years, electrospun silica nanofibers have been obtained by combination of the electrospinning process and sol–gel methodology without containing any binder or organic gelling agent that promotes spinnability [35]. By controlling parameters such as, viscosity of tetraethoxysilane (TEOS) solution, ethanol concentration and degree of crosslinking, it is possible to produce homogeneous, beadless silica nanofibers. Herein, silica nanofibers (SiNF) were obtained via an electrospinning process using a TEOS solution containing ethanol, deionized water and HCl. The average thickness of the SiNF mat after drying was about 20 μm.

**Figure 2:**
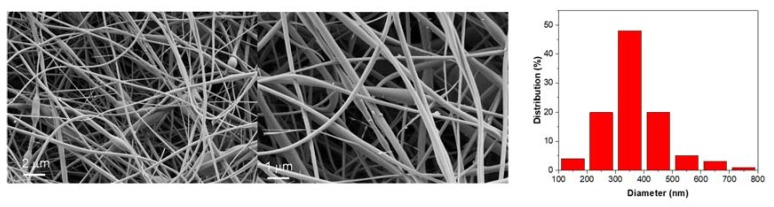
SEM images of SiNF at different magnifications and thickness distribution of SiNFs.

The morphology of silica nanofiber mats was evaluated by SEM and as shown in Figure 2, the nanofibers were interlaced with each other forming a 3D multi-layered interpenetrating fibrous network (Figure S1 in Supplementary Materials). The nanofibers showed an average diameter of 350 nm bearing oval beads with a diameter of about 500 nm, which may be formed driven by the surface tension, as previously reported [36].

**Figure 3:**
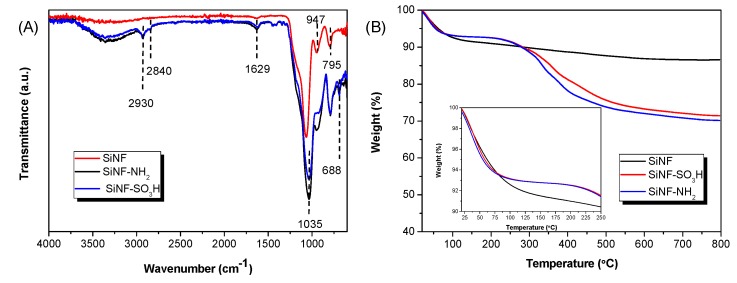
(**A**) ATR-FTIR spectra and (**B**) thermal stability of silica nanofiber mats.

The successful functionalization of the SiNF mats was confirmed by X-ray photoelectron spectroscopy (XPS, JEOL Ltd, Garden City, United Kingdom) and Fourier-transform infrared spectroscopy (FTIR). XPS is a widely used surface-sensitive quantitative spectroscopic technique for the measurement of the elemental composition within a surface or material [37,38,39]. Unmodified SiNF mats showed peaks corresponding to Si2s, Si2p and O1s in the XPS spectra. After APTES modification, the characteristic peaks of N1s and C1s at 400 and 285 eV, respectively, were also visible in the XPS spectra due to the formation of the organic layer. Regarding the acidic SiNF–SO_3_H mats, the characteristic XPS peaks of S2p and C1s at 167 and 285 eV, respectively, confirmed the successful modification with acidic –SO_3_H groups (Figures S2–S4 in Supplementary Materials). The ATR–FTIR spectra of nanofiber mats is displayed in Figure 3A and shows one characteristic band near 1035 cm^−1^ for all the mats, resulting from the Si–O–Si stretching. After APTES modification, the grafting of –NH_2_ groups gave rise to two characteristic bands at 947 and 1629 cm^−1^ for silica nanofibers containing the basic –NH_2_ group, which were assigned to the out-of-plane bending and scissoring vibration of N–H, respectively [40]. For silica nanofibers containing the acidic –SO_3_H group, the characteristic band corresponding to O=S=O vibration at 1055 cm^−1^ was overlapped by the broad band appearing at 1035 cm^−1^ [41]. 

Thermogravimetric analysis performed on nanofiber mats show an elevated stability of these inorganic materials. TGA thermograms of the different nanofiber mats are compared in Figure 3B. After heating at 100 °C, mats showed a weight loss of 6–7%, which may be attributed to the release of moisture in the membrane and also to the self-condensation reaction of the remaining silanol groups from TEOS [42]. Upon surface functionalization with APTES or MPTMS, both SiNF–NH_2_ and SiNF–SO_3_H mats displayed a similar thermal degradation behavior, with a 15% weight loss between 300 and 500 °C, which can be attributed to the degradation of the organic groups from the attached organic layers. These results show the high thermal stability of the SiNF mats and its further application in as reinforcing fillers in the preparation of mixed matrix membranes for HT-PEMFCs.

### 3.2. Preparation and Characterization of PBI Composite Membranes Containing Silica Nanofibers

**Figure 4:**
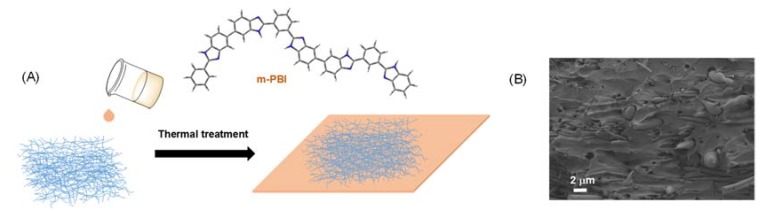
(**A**) Schematic representation of preparation of composite membrane containing SiNF, and (**B**) SEM image of composite membrane containing SiNF.

Next, we prepared composite PBI membrane containing the nanofiber mats containing the different functional groups. To this end, the functionalized nanofiber mats were impregnated in a 10 wt % PBI solution to yield a composite membrane after a drying process with a final thickness around 150 μm, controlling the nanofiber loading of 10 wt % of the final membrane weight (Figure 4). The thickness of all composite membranes was uniform across the whole surface. A representative example of a SEM micrograph of fracture surface in liquid nitrogen of composite membrane is given in Figure 4B. As can be seen, the interfacial debonding and the pull-out of fibers in the fractured composite is clearly visible and also, the presence of holes around the nanofibers. These observations are representative of a poor adhesion between the nanofiber mat and the PBI matrix, which might be attributed to the low interaction of hydrophilic groups from the SiNF mat and the hydrophobic polymer chains. However, it is expected that the presence of sites of cohesive matrix fracture may contribute to an overall improvement in the strength of the composite.

The incorporation of silica nanofibers into the PBI matrix was also analyzed by FT-IR spectroscopy (Figure S5 in Supplementary Materials). PBI spectrum shows three characteristic peaks at 3415, 3140, and 3065 cm^−1^ attributed to non-hydrogen bonded and free N–H groups, self-associated hydrogen-bonded N–H groups and stretching modes of aromatic C–H groups, respectively [43]. Additionally, sharp bands corresponding to the Si–O–Si stretching of NF mats at 1035 cm^−1^ were visible, indicating the presence of the NF mat in the polymer matrix. 

The water uptake (WU) behavior of the membrane is an important parameter to be considered in PEMFC applications, as high values of WU are highly demanded for improving the formation of the hydrophilic domain, which is responsible of the proton conductivity and contributes positively to the presence of vehicles for proton transport through the membrane [44]. On the contrary, an elevated WU value may lead to an excessive swelling and produce undesired mechanical degradation. Therefore, a proper balance between the WU and swelling ratio (SR) is crucial for the future operation of the polyelectrolyte in a membrane electrode assembly (MEA). The WU and SR values for the different composite membranes are shown in Table 1. As observed, the addition of unmodified SiNFs to the polymeric matrix increased WU and SR values in comparison to the pristine PBI membrane (from 7% to 27% and from 9% to 36%, respectively). For composite membranes containing acidic or basic functionalized SiNFs, a significant enhancement of both parameters was observed, which might be attributed to the hydrophilic character of the functional groups in the modified SiNFs [45]. In this regard, amino and sulfonic groups can form H-bonds with water molecules and therefore, contribute to an enhancement of the WU.

**Figure 5:**
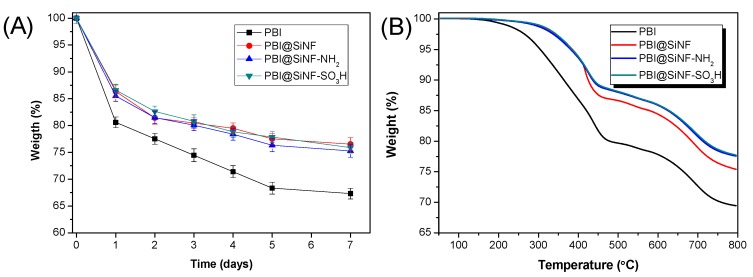
(**A**) Oxidative stability after Fenton′s test and (**B**) thermal stability of composite membranes containing SiNFs.

Oxidative stability is another important parameter under consideration in the fabrication of PEMFCs, as it affects the long−term operation and the performance of the polymeric membrane. In this regard, Fenton′s test was used to investigate the chemical stability of the composite membranes containing nanofiber mats [46]. For this purpose, the oxidative stability of the membranes was studied by immersion in freshly prepared Fenton’s reagent (3% H_2_O_2_ solution containing 4 ppm Fe^2+^) at a temperature of 70 °C along different periods of time. Then, the oxidative stability of the membranes was calculated by their weight loss. As shown in Figure 5A, all composite membranes containing nanofiber mats displayed a similar oxidative degradation pattern, which showed a better stability than that observed for the pure PBI membrane. The enhanced oxidative resistance may be attributed to the hydrogen bond interactions between the polar groups on silica surface and the imidazole groups of PBI chains, as observed for similar systems [47].

Figure 5B shows the thermograms of PBI and composite PBI membranes containing the nanofiber mats (performed under nitrogen atmosphere). For the neat PBI sample, a first weight loss around 2–7% was observed in the interval ranging from room temperature to 250 °C. This degradation step was followed by a 20% weight loss at 450 °C. In the case of PBI membranes containing the nanofiber mats, these composite membranes showed higher thermal stability than the pure PBI membrane in the range from 50 to 300 °C, with only a 10% weight loss at 450 °C. Among the three composite PBI membranes, those containing functionalized nanofibers, namely PBI@SiNF–NH_2_ and PBI@SiNF–SO_3_H, were more stable at elevated temperatures (above 400 °C) than the non-functionalized PBI@SiNF, as also observed for similar mixed matrix membranes [48]. The enhanced thermal stability displayed for the composite PBI membranes containing silica nanofibers shows that the as-prepared materials possess an adequate thermal stability for its future application as proton exchange membrane fuel cells capable to operate at intermediate or elevated temperatures.

The tensile properties of the membranes were determined from stress–strain curves obtained with a universal testing machine (Shimadzu AGS-X, Shimadzu, Tokyo, Japan) at a crosshead rate of 10 mm∙min^−1^ at room temperature. For that, samples of 30 mm × 6 mm and with a thickness 150 μm thick (five samples of each type of membranes) were tested and the average results with standard deviation are given in Table 2. For a proper comparison, the Young′s modulus, tensile stress and strain at break values of the pure PBI are included. The weight percentage of silica nanofiber in the composites was determined through weight loss by thermogravimetric analysis (TGA). The final percentage of silica nanofibers (SiNFs) in all the samples was around 10 wt %. The incorporation of silica nanofibers into the PBI matrix led to an increase in mechanical resistant properties (Young′s modulus and tensile stress). The major increase in mechanical resistant properties was observed for PBI@SiNF–NH_2_ sample, where the modulus and tensile stress increased about 20% compared with neat PBI sample. These results may be attributed to the strong hydrogen bonding interaction between NH groups of PBI and SiNF, which can affect the mobility of chain segments [49]. From a structural point of view, the addition of silica nanofiber mat perturbs the normal polymer flow and restricts the mobility of polymer chains in PBI. Furthermore, the inorganic nature of SiO_2_ nanofibers, whose main characteristic is their low elongation to break, as well as the possible defects introduced during the sample preparation, contributes to reduce the ductility in the composite membranes. However, this slight reduction in flexibility does not have a significant effect for its use as proton exchange membrane in fuel cells, as observed in other reported studies [50,51].

### 3.3. Conductivity Measurements of PBI Composite Membranes Containing Silica Nanofibers

**Figure 6:**
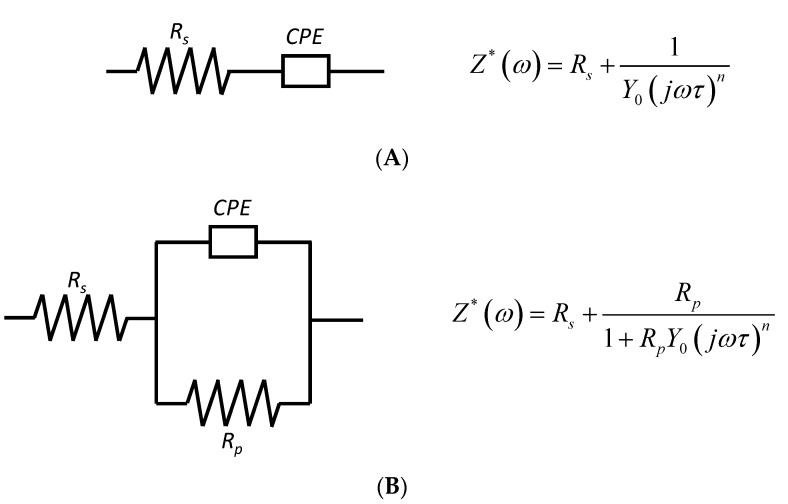
Equivalent circuits used to study the behavior of the PBI composite membranes containing silica nanofillers analyzed in (**A**) dry and (**B**) wet conditions.

The study of proton conductivity is an important feature to evaluate the potential performance of novel PEMs. In this study, DC conductivity (σ*_dc_*) was calculated from the impedance spectroscopy measurements obtained using a Novocontrol broadband dielectric spectrometer (Figure S6 in Supplementary Materials) with a blocking electrode configuration. Under an alternating electric field, the response of the charges or dipoles linked to chain segments of the polymer matrix, as well as the ion movement, is dependent of the physical state of the system. When using a blocking electrode configuration, the polarization process is not well defined by a single relaxation time (Debye process), and consequently, a distribution of relaxation times (DRT) is required. In general, the experimental complex impedance can be represented by means of equivalent circuits consisting in a polarization resistance in series or parallel, with a constant element phase (CPE) having an admittance given by Y* = *Y*_0_(jωτ)*^n^*, where *n* is a frequency independent parameter, generally in the range 0 < *n* ≤ 1, j is the imaginary unit (j^2^ = −1), and Y_0_ is given in Ω^−1^ [52], as displayed in Figure 6.

**Figure 7:**
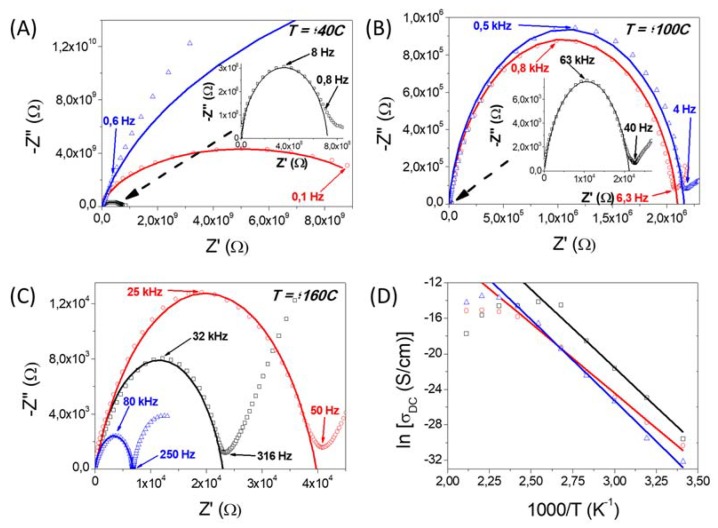
(**A**–**C**) Cole-Cole plots of the complex impedance measured in dry conditions at different temperatures for the samples: (**☐**) PBI@SiNF, (**⚪**) PBI@SiNF–NH_2_ and (**Δ**) PBI@SiNF–SO_3_H. (**D**) The solid line represents the fitting to the equivalent circuit models shown in Figure 6A. The inset in plots at 40 and 100 °C corresponds to the small arc indicated by the arrow and observed for the composite membrane PBI@SiNF.

**Figure 8:**
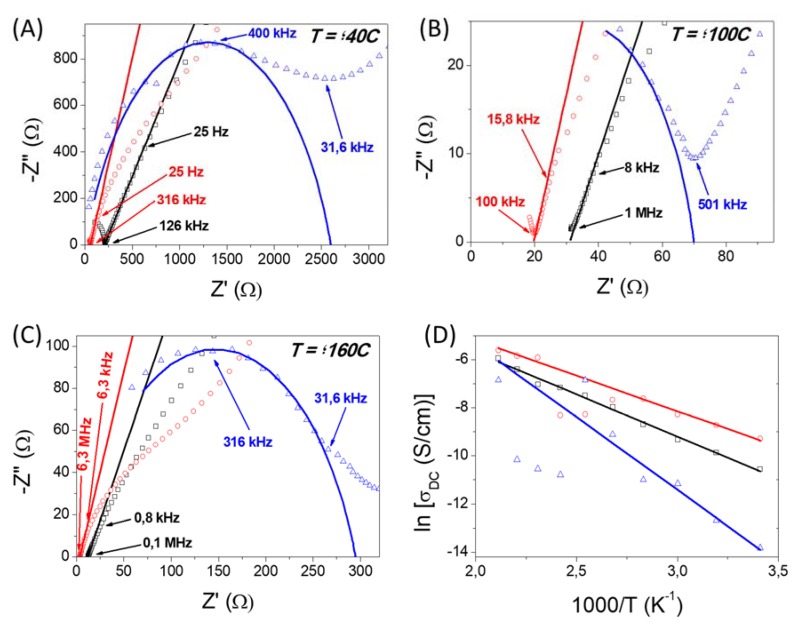
(**A**–**C**) Cole-Cole plots of the complex impedance measured in wet conditions at different temperatures for the samples: (**☐**) PBI@SiNF, (**⚪**) PBI@SiNF–NH_2_ and (**Δ**) PBI@SiNF–SO_3_H. (**D**) The solid line represents the fitting to the equivalent circuit models as shown in Figure 6B.

Figure 7 and Figure 8 show the Cole-Cole plots (also known as Nyquist diagrams) of complex impedance for the composite PBI membranes containing the nanofiber mats at different temperatures (40, 100 and 160 °C) under dry and wet conditions, respectively. In these graphical representations, the real and imaginary parts of the complex impedance, Z′ and Z′′ (both given in Ω), respectively, were fitted using the equivalent circuits displayed in Figure 6. Under dry conditions, the Cole-Cole plots was fitted using a single equivalent circuit model consisting on a resistive element and a constant phase element (CPE). In our study, the proton conductivity measurements over membranes containing different SiO_2_ nanofibers were initially performed under wet conditions at the temperature range between 20 and 120 °C in order to remove all water present in the membrane. After, these measurements, the sampled was cooled down 0 °C, and measurements under dry conditions were performed from to 20 and 200 °C. As observed in Figure 7A–C, which displays the Cole-Cole plots for composite membranes containing the three nanofiber mats measured under dry conditions, all spectra exhibited a large arc at high frequencies. Notice that at these frequencies, the intercept is correlated with the DC-conductivity of the composite membranes. For example, the resistance values at 160 °C were 2300, 6800 and 40,000 Ω, for PBI@SiNF, PBI@SiNF–SO_3_H and PBI@SiNF–NH_2_ composite membranes, respectively. When increasing the temperature, the arc intersect was shifted to lower frequencies. This effect was different when working under wet conditions, where a decrease around three orders of magnitude was observed for the real part of the impedance for each sample when increasing temperature from 40 to 100 °C, and two orders of magnitude when increasing from 100 to 160 °C. 

As mentioned above, the DC conductivity at a given temperature was obtained from the Nyquist diagram by fitting the obtained semicircles using the corresponding equivalent circuit illustrated in Figure 6 (Tables S1 and S2 in Supplementary Materials). From the fit, the DC conductivity can be calculated form the expression σ = *L*/(*A*·*R*), where *R* is the resistance of the composite membrane represented by the arc intercept in the real axis, *L* is the sample thickness, and *A* is the area of each disk sample sandwiched between the two gold electrodes. Notice that this procedure is an indirect method, because the criteria needs to be considered, in this case, the selected equivalent circuit, in order to obtain the value of the resistance. On the other hand, the fit when operating under wet conditions was modelled using a parallel combination of a resistive element and a CPE, which accounts the interfacial phenomena in the membrane-electrode interface. On the contrary, it was observed that the resistance under dry conditions, given by the intercept of the arc with the axis Z’, yielded higher values of the impedance in comparison with the measurements under wet conditions for the same sample.

Following this procedure, the obtained resistances at 100 °C and under wet conditions for the composite membranes PBI@SiNF−NH_2_, PBI@SiNF−SO_3_H and PBI@SiNF were 20, 40 and 70 Ω, respectively (Figure 8A–C). From these resistance values, proton conductivities of 1.10 × 10^−4^, 4.62 × 10^−4^ and 3.44 × 10^−4^ S·cm^−1^, were obtained for PBI@SiNF−NH_2_, PBI@SiNF−SO_3_H and PBI@SiNF membranes, respectively. In all cases, a conductivity improvement of several orders of magnitude was observed when comparing these composite membranes with the pristine PBI membrane, which showed low conductivity values around 10^−5^ S·cm^−1^.

The impedance responses are typical of electrolytes whose contribution is generally related to the bulk resistance, and generally, only a minor contribution is associated to the grain boundary resistance of the nanofibers. The intercept on the real-axis exhibiting bulk resistance varies under wet conditions between 200 and 12 Ω, for the composite PBI@SINF, from 55 to 3.5 Ω, for PBI@SiNF−NH_2_ and from 700 to 66 Ω, for PBI@SiNF−SO_3_H membrane. The comparison of proton conductivity for composite membranes is very significant, and maximum values were obtained for the mixed matrix membrane containing nanofibers with basic amino groups, with values up to 0.004 S·cm^−1^ at 200 °C. This conductivity is higher than that observed for undoped pristine PBI membranes [53]. However, under the same operating conditions SiO_2_ nanofibers containing acidic sulfonic groups reached values lower therefore the character basic or acid in the nanofiber mats is very influenced in their properties. Hence, proton conductivity is strongly dependent on the number of proton acceptors and donors present in the nanofiber mats. Furthermore, we conclude that the order of capacity for proton transfer is −NH_2_ > −OH > −SO_3_H. Notice from Figure 8 that composite membrane PBI@SiNF–SO_3_H have an arc in the plots at temperatures of 40, 100 and 160 °C; while in the other two composite membranes, namely PBI@SiNF and PBI@SiNF–NH_2_, this arc it is not present. Therefore, it is worth mentioning that the equivalent circuit used to calculate the resistance of the bulk of the PBI@SiNF–SO_3_H membrane was different to that used for the others membranes under study.

The ionic conductivity largely depends on the porous structure that entraps liquid electrolytes and consequently, the formation of pores in membranes is critical when obtaining proper channel of ionic conduction [54,55,56,57]. Under dry conditions, the obtained ionic conductivities of all samples under study were higher than 10^−3^ S·cm^−1^ at high temperatures (up to 180 °C), which might be attributed to interaction of the interlaced nanofiber structure with the polymeric matrix. In addition, the incorporation of basic SiO_2_–NH_2_ nanofibers in contrast with the acidic SiO_2_–SO_3_H was reflected in an increase of the conductivity reaching values as high as 3.6 × 10^−3^ S·cm^−1^. The enhancement of ionic conductivity in composite polymer electrolytes has been attributed mainly to the decrease of the polymer crystallinity in the presence of the inorganic particles, and also to the Lewis acid–base type interactions between the inorganic particles and the electrolyte polar groups, being more favorable with basic rather than with acidic groups for PBI-based polymers. These effects have also been observed in composite nanofiber membranes based on poly(vinylidene fluoride) (PVDF) with different silica contents [58,59] and PVA membranes with GO [60]. 

When comparing the obtained values for the PBI mixed matrix membranes with the previously reported values for other commonly used composite membranes [61,62,63], such as Nafion or SPEEK, those for PBI are generally one order of magnitude lower as they operate at elevated temperatures, where anhydrous conditions are reached at high temperatures. It is worth mentioning that operating temperatures in most reports found in literature do not exceed 80–90 °C, which hampers a proper comparison. However, a comparison with other PBI membranes reveals that generally proton conductivity studies for PBI membranes are performed after acid doping; however, acid leaching studies and stability tests after a few operating cycles are often omitted [64]. The described composite PBI membrane containing silica nanofiber mats notably increased the proton conductivity with respect to pristine PBI membrane, which has a low proton conductivity in the absence of any acid filler, but the values are still far from those of Nafion membranes; only doping with concentrated phosphoric acid can yield values in the range of the aforementioned PFSA polymers.

Figure 7D and Figure 8D display the Arrhenius plot, showing temperature dependence of the protonic conductivity. As a general overview, an increase of the conductivity of the PBI composite with temperature was observed. In general, the conductivity behavior with temperature follows the Arrhenius equation given by the expression ln σ*_dc_* = ln(σ_0_) − (*E*_act_/R*T*), where σ*_dc_* is the proton conductivity of the composite membrane (S·cm^−1^), σ_0_ is a pre-exponential factor (S·cm^−1^), *E*_act_ is the activation energy (kJ·mol^−1^), R is the ideal gas constant (8.314 J·mol^−1^·K^−1^) and T is the temperature (K). From the ln (σ*_dc_*) vs. (1000/*T*) graphical representation, the activation energy of a given sample can be obtained from the slope of the linear fit (Table 3). As observed, the *E*_act_ values of the composite membranes under wet conditions were lower than that for pure PBI membrane, which is associate to a facile proton mobility, as the nanofiber acts as a carrier-bridge for protons and consequently, the process demands less energy. As also inferred from Table 2, the activation energy follows the trend: *E*_act_ (PBI@SiNF–SO_3_H) > *E*_act_ (PBI@SiNF) > *E*_act_ (PBI@SiNF–NH_2_), which shows the conduction process is more favorable in the presence of basic groups. The values for membranes containing basic nanofiber mats are quite similar to those found for activation energies of nanofiber-reinforced membranes based on Nafion [25], polystyrene [65], SPEEK [66], poly(vinyl butyral) (PVB) [67], polysulfone (PSU) [68], sulfonated polyimide (PI) [69] and alginate/carrageenan membranes [70]. Regarding proton conduction processes in polyelectrolytes and polymeric membranes, two main pathways are generally accepted. On one hand, the Grotthuss mechanism explains the conductivity by means of the interaction of protons through the jump between a hydrogen bond network of N−H groups, both from the PBI and from the functionalized groups of the nanofiber mats. On the other hand, protons can move via the vehicle mechanism through the hydroxyl, amine or sulfonic groups from the different nanofiber functionalization and imidazole groups present in the PBI, which may interact with water molecules, promoting the proton conductivity. It is also noteworthy that all membranes containing nanofiber mats have lower activation energies than pristine PBI membranes and also lower than hydrated membranes of SPEEK reinforced membranes which values are compress between 9.5 and 48 kJ·mol^−1^ depending on water content and acid dopant concentration [71,72,73]. On the other hand, activation energies under dry conditions support the proton conduction is mainly due according to a vehicular mechanism, as inferred from the calculated activation energies, with values higher than 55 kJ·mol^−1^.

## 4. Conclusions

In summary, this paper shows the use of silica nanofibers obtained by the electrospinning method as reinforcing fillers in the preparation of mixed matrix membranes based on PBI polymer. The as-prepared nanofiber mats could be chemically modified to give acidic and basic functionalized nanofibers, which showed to be efficient conductive fillers in the preparation of mixed matrix membranes. The incorporation of these nanofillers in the PBI matrix improves the chemical and thermal stability of the mixed matrix membranes, as well as the proton conductivity. In this regard, conductivities up to 4 mS·cm^−1^ at elevated temperatures (200 °C) were obtained for the corresponding composite membrane containing basic groups; however, composite membranes containing acidic groups did not improve the proton conductivity. Hence, conductivity was found to be strongly dependent on the acid-base properties of proton acceptors and donors present in the nanofiber mat. These results show that an oriented chemical modification of silicon nanofibers has a representative effect in the proton conductivity of composite membranes containing the aforementioned nanofibers. This fine-tuning facilitates the optimization process and opens the door for the future development of high-temperature electrolytes containing functionalized nanofibers in the fabrication of electrochemical devices for energy applications.

## Figures and Tables

**Table 1:** Water uptake (WU) and swelling ratio (SR) values for the PBI composite membranes.

Membrane	WU (%)	SR (%)
PBI	7 ± 1	9 ± 2
PBI@SiNF	27 ± 3	36 ± 3
PBI@SiNF–NH_2_	34 ± 3	48 ± 2
PBI@SiNF–SO_3_H	36 ± 2	46 ± 2

**Table 2:** Mechanical properties of the PBI composite membranes containing SiNFs.

Membrane	Young′s Modulus (GPa)	Tensile Stress (MPa)	Strain at Break (%)
PBI	2.6 ± 0.5	97 ± 4	27 ± 4
PBI@SiNF	2.9 ± 0.3	114 ± 2	11.6 ± 0.9
PBI@SiNF–NH_2_	3.2 ± 0.1	119 ± 3	10.3 ± 1.2
PBI@SiNF–SO_3_H	2.7 ± 0.3	110 ± 2	13.1 ± 0.7

**Table 3:** Activation energies for the PBI composite membranes in wet and dry conditions.

Membrane	*E*_act(wet)_ (kJ·mol^−1^)	*E*_act(dry)_ (kJ·mol^−1^) ^a^
PBI	55.6 ± 0.8	75 ± 3
PBI@SiNF	12.7 ± 0.4	72 ± 3
PBI@SiNF–NH_2_	10.7 ± 0.3	56 ± 2
PBI@SiNF–SO_3_H	25 ± 1.5	123 ± 10

^a^ The activation energy was calculated for the temperature interval 20–90 °C.

## Supplementary Materials

The following are available online at https://www.mdpi.com/2073-4360/11/7/1182/s1, Materials and methods. Figure S1: XPS N1s spectra of unmodified SiNF, Figure S2: XPS N1s spectra of SiNF after APTES modicification, Figure S3: XPS S2p spectra of acidic SiNF, Figure S4: SEM image of SiNF, Figure S5. ATR-FTIR spectra of PBI@SiNF, PBI@SiNF–NH_2_ and PBI@SiNF–SO_3_H; Figure S6. Schematic illustration of the Novocontrol Concept 80, Table S1: Conductivity values obtained from the Nyquist diagram for PBI and different PBI@SiNF under wet conditions; Table S2: Conductivity values obtained from the Nyquist diagram for PBI and different PBI@SiNF under dry conditions.
