# Supplementary material for: Proton Conductivity through Polybenzimidazole Composite Membranes Containing Silica Nanofiber Mats
Source: Polymers (Basel). 2019 Jul 14;11(7):1182. doi: 10.3390/polym11071182 (PMC6680558; doi:10.3390/polym11071182)
Supplement: Supplementary file 1 [file polymers-11-01182-s001.pdf]

Supporting Information

Proton Conductivity through Polybenzimidazole Composite Membranes Containing Silica Nanofibrous Mats

Jorge Escorihuela [1,2,*], Abel García-Bernabé [1], Álvaro Montero [1], Andreu Andrio [3], Óscar Sahuquillo [4], Enrique Giménez [4] and Vicente Compañ [1,*]

[1] Departamento de Termodinámica Aplicada (ETSII) Universitat Politècnica de València, Camino de Vera s/n, 46022 Valencia, Spain; agarciab@ter.upv.es (A.G.-B.); almonter@upvnet.upv.es (A.M.)
[2] Departament de Química Orgànica, Universitat de València, Av. Vicent Andrés Estellés s/n, Burjassot, 46100 Valencia, Spain;
[3] Departament de Física Aplicada. Universitat Jaume I, 12080 Castelló, Spain; andrio@uji.es;
[4] Instituto de Tecnología de Materiales, Universitat Politècnica de València, Camino de Vera s/n, 46022 Valencia, Spain; ossana@upvnet.upv.es (O.S.); enrique.gimenez@mcm.upv.es (E.G.)
* Correspondence: escorihu@uji.es (J.E.); vicommo@ter.upv.es (V.C.); Tel.: +34-96-387-9328

1. Materials and Methods

1.1. Characterization

1.1.1. Morphology analysis.

Scanning electron microscopy (SEM) images were acquired on a field emission scanning electron microscope (FE–SEM) model Ultra 55 (Zeiss, Oberkochen, Germany) operating at 5 kV with energy-dispersive X-ray (EDX) spectroscopy. Electron micrographs were obtained using a Jeol JEM–1010 high resolution microscope (JEOL Ltd, Garden City, United Kingdom).

1.1.2. X-ray Photoelectron Spectroscopy (XPS).

The XPS analysis of surfaces was performed using a JPS-9200 photoelectron spectrometer (JEOL Ltd, Garden City, United Kingdom). Survey and highresolution spectra were obtained under UHV conditions using monochromatic Al Kα X-ray radiation at 12 kV and 20 mA, and an analyzer pass energy of 50 eV for wide scans and 10 eV for narrow scans. The X–ray incidence angle and the electron acceptance angle was 10° to the surface normal. The takeoff angle φ (angle between sample and detector) of 80° is defined to a precision of 1°. The intensity of the XPS core-level electron was measured as the peak area after standard background subtraction according to the linear procedure. All XPS spectra were evaluated using the Casa XPS software (version 2.3.19, Casa Software Ltd, Las Vegas, NV, USA).

1.1.3. Infrared spectrocopy.

Attenuated total reflection Fourier transform infrared (ATR-FTIR) spectra of the membranes were recorded on a Jasco FT-IR spectrometer FT/IR-6200 Series (Jasco Spain, Madrid, Spain) with a 4 cm^{-1} resolution between 400 and 4000 cm^{-1}.

1.1.4. Thermal stability.

Thermogravimetric analysis (TGA) was performed on a TGA Q50 thermogravimetric analyzer TGA Q50 (Waters Cromatografia, S.A., Division TA Instruments, Cerdanyola del Valles, Spain). The samples (5–10 mg) were weighed in platinum crucibles and were heated under nitrogen atmosphere (60 $mL \cdot min^{-1}$) from room temperature to 800 °C at a heating rate of 10 $°C \cdot min^{-1}$. For the surface area and porosity analysis, the solid or membrane was dried in a vacuum oven at 100 °C for 5 h and activated at 100 °C for 12 h on a SmartVacPrep instrument (Micromeritics Instrument Corporation, Norcross, GA, USA).

1.1.5. Water uptake.

The water uptake (WU) of the membrane was calculated by the following equation: WU (%) = $(W_{wet} - W_{dry})/W_{dry} \times 100$; where W_{wet} and W_{dry} refer to the membrane's weight after its immersion in deionized water for at least 48 h at room temperature and the membrane's weight after drying at 120 °C for at least 24 h, respectively.

1.1.6. Oxidative stability.

The oxidative stability (OS) of the membranes was investigated by immersing the membranes in Fenton's reagent (3% H_2O_2 solution containing 4 ppm Fe^{2+}) at 70 °C. The samples were collected by filtering and rinsed with deionized water several times, then dried at 120 °C for 5 h in a vacuum oven. Next, the degradation of the membranes was evaluated by their weight loss by using the following equation: OS (%) = $((W_1 - W_2)/W_1) \times 100$; where W_1 is the weight of the dried membrane before the Fenton test and W_2 is the weight of the dried membrane after the Fenton test.

1.1.7. Mechanical properties.

The tensile tests from each thin-film composite membrane were performed using a Shimadzu AGS-X Universal Testing Machine (Izasa Scientific, Madrid, Spain). The mechanical parameters were determined from the average of five samples. For all of the tests, a tensile speed of 5 $mm \cdot min^{-1}$ and a load cell of 500 N were used.

1.1.8. Proton conductivity.

The proton conductivity measurements of the membranes in the transversal direction were performed in the temperature range between 0 and 200 °C by electrochemical impedance spectroscopy (EIS) in the frequency interval of 0.1 to 10 MHz, applying a 0.1 V signal amplitude. A broadband dielectric spectrometer (Novocontrol Technologies, Hundsangen, Germany) integrated with an SR 830 lock–in amplifier with an Alpha dielectric interface was used. The membranes were previously immersed in deionized water and the thickness was measured afterwards using a digital micrometer, taking the average measurements at different parts of the surface. Then, the membranes were placed between two gold electrodes coupled to the spectrometer. Initially, in the first scan, the temperature was gradually increased from 20 to 200 °C in steps of 20 °C (named as wet conditions in the manuscript) and the dielectric spectra were collected at each step. During the second cycle of temperature scan (named as dry conditions in the manuscript), the dielectric spectra were collected at each step from 20 to 200 °C, in steps of 20 °C.

2. Supplementary Figures

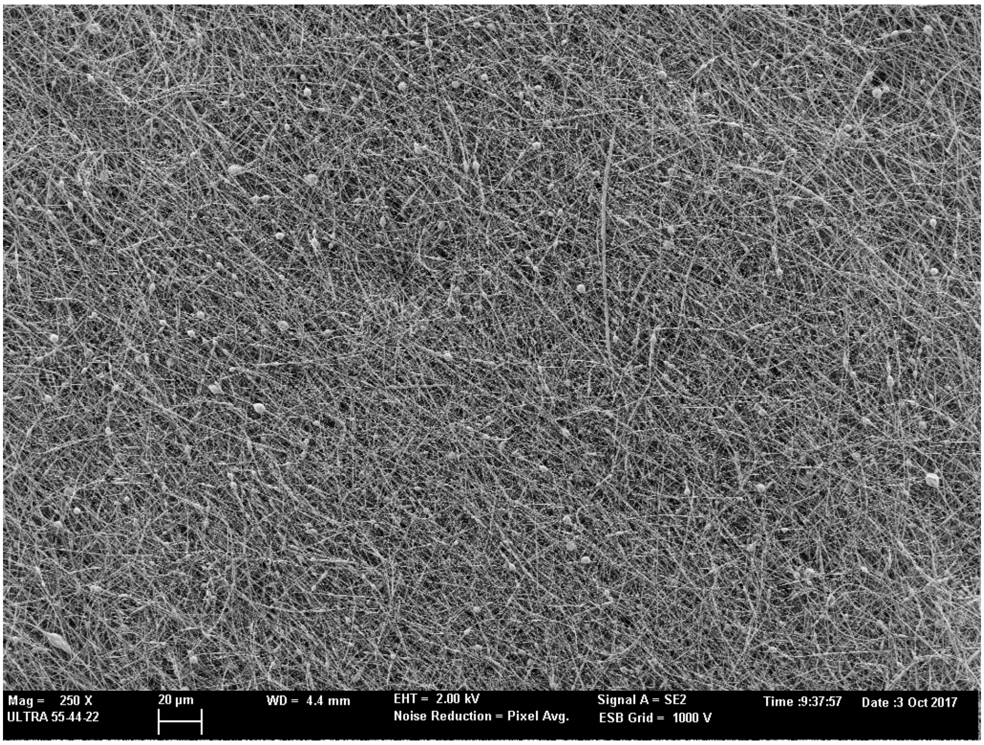

Figure S1. SEM image of SiNF.

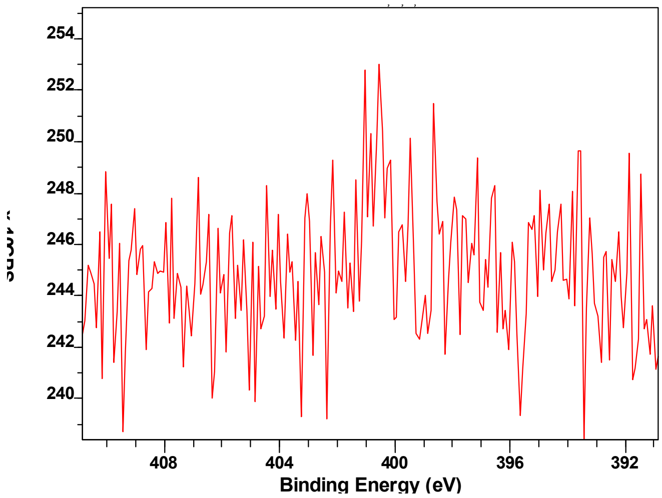

Figure S2. XPS N1s spectra of unmodified SiNF.

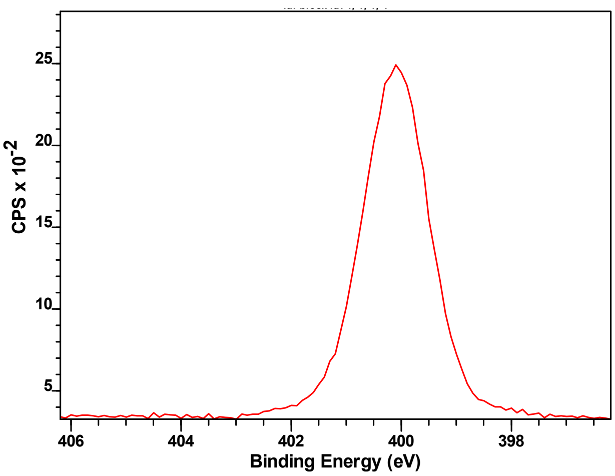

Figure S3. XPS N1s spectra of SiNF after APTES modicification.

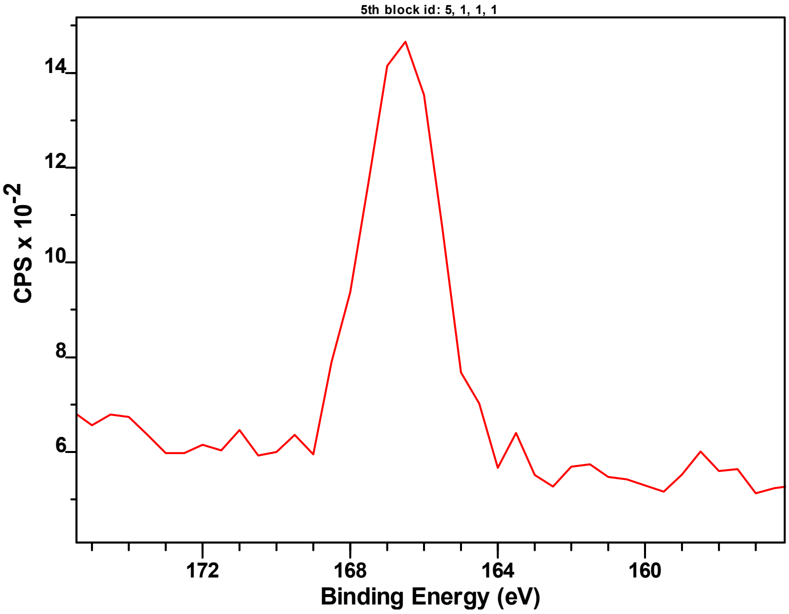

Figure S4. XPS S2p spectra of acidic SiNF.

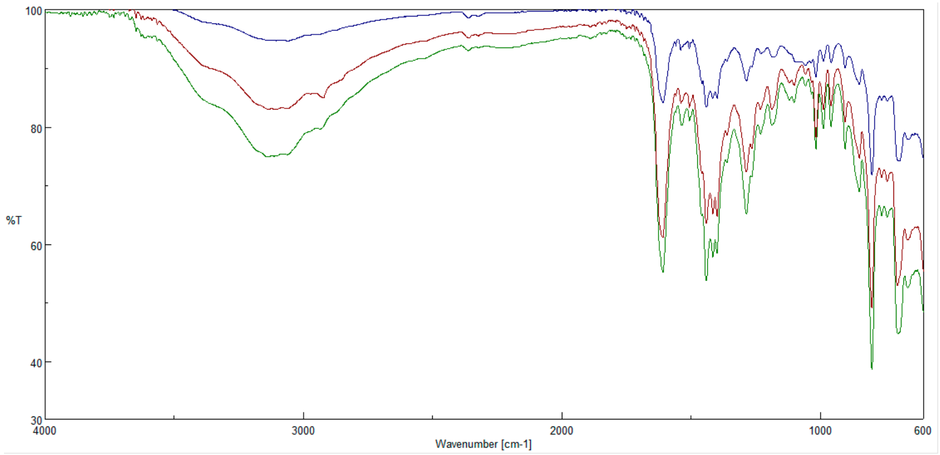

Figure S5. ATR-FTIR spectra of PBI@SiNF (**green**), PBI@SiNF–NH_2 (**blue**) and PBI@SiNF–SO_3H (**red**).

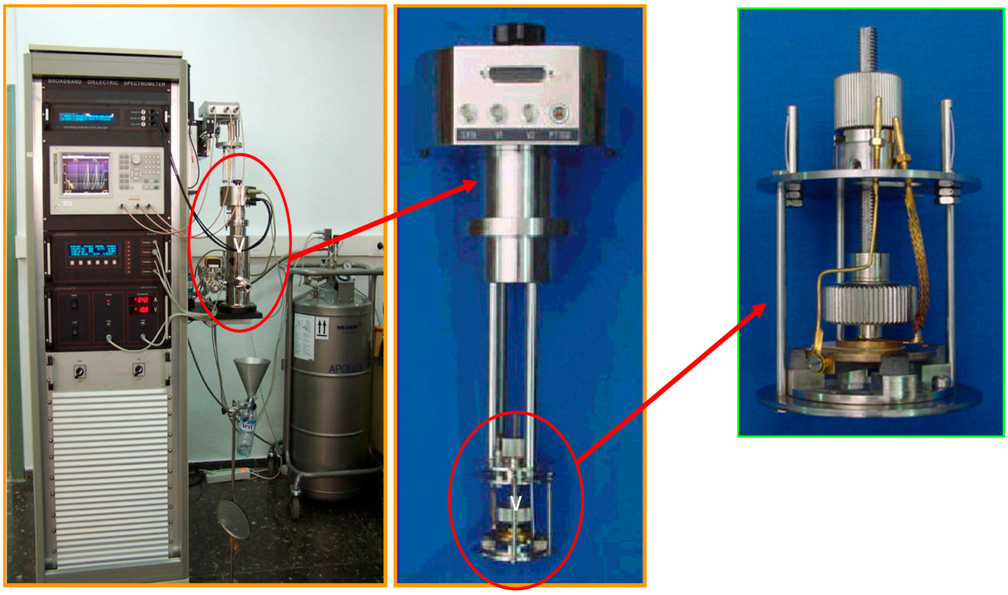

Figure S6. Schematic illustration of the Novocontrol Concept 80.

3. Supplementary Tables

Table S1. Conductivity values obtained from the Nyquist diagram for PBI and different PBI@SiNF under wet conditions.

T (°C)	PBI	PBI@SiNF	PBI@SiNF–NH_2	PBI@SiNF–SO_3H
20	7.52×10^{-11}	2.60×10^{-5}	9.27×10^{-5}	9.88×10^{-7}
40	3.22×10^{-10}	5.20×10^{-5}	1.61×10^{-4}	3.08×10^{-6}
60	9.34×10^{-10}	8.81×10^{-5}	$2{,}56 \times 10^{-4}$	1.43×10^{-5}
80	1.26×10^{-9}	1.70×10^{-4}	4.89×10^{-4}	1.67×10^{-5}
100	1.14×10^{-9}	3.44×10^{-4}	4.62×10^{-4}	1.10×10^{-4}
120	7.18×10^{-10}	5.55×10^{-4}	2.56×10^{-4}	1.06×10^{-3}
140	1.24×10^{-10}	7.78×10^{-4}	2.45×10^{-4}	2.05×10^{-5}
160	1.36×10^{-11}	8.89×10^{-4}	2.71×10^{-3}	2.61×10^{-5}
180	5.02×10^{-12}	1.67×10^{-3}	2.92×10^{-3}	3.85×10^{-5}
200	3.18×10^{-12}	2.60×10^{-3}	3.54×10^{-3}	1.06×10^{-3}

Table S2. Conductivity values obtained from the Nyquist diagram for PBI and different PBI@SiNF under dry conditions.

T (°C)	PBI	PBI@SiNF	PBI@SiNF–NH_2	PBI@SiNF–SO_3H
20	1.2×10^{-14}	1.42×10^{-13}	8.32×10^{-14}	2.34×10^{-13}
40	1.2×10^{-13}	1.48×10^{-11}	9.30×10^{-13}	1.10×10^{-13}
60	3.9×10^{-12}	3.95×10^{-10}	149×10^{-11}	9.45×10^{-12}
80	8.5×10^{-12}	8.74×10^{-9}	2.43×10^{-10}	1.71×10^{-10}
100	1.5×10^{-11}	5.02×10^{-7}	4.40×10^{-9}	3.54×10^{-9}
120	0.9×10^{-11}	7.11×10^{-7}	4.78×10^{-8}	6.25×10^{-8}
140	------	4.59×10^{-7}	1.31×10^{-7}	4.35×10^{-7}
160	------	4.64×10^{-7}	2.24×10^{-7}	1.13×10^{-6}
180	------	1.60×10^{-7}	2.88×10^{-7}	1.39×10^{-6}
200	------	1.96×10^{-8}	2.65×10^{-7}	6.49×10^{-7}
